# More Than Meets the Eye: The Contributions of John Dalrymple (1803-1852)

**DOI:** 10.7759/cureus.1366

**Published:** 2017-06-19

**Authors:** Joy MH Wang, Bryan Edwards, Gabrielle G Tardieu, Marios Loukas, Rod J Oskouian, R. Shane Tubbs

**Affiliations:** 1 Department of Anatomical Sciences, St. George's University School of Medicine, Grenada, West Indies; 2 Department of Anatomical Sciences, St. George's University School of Medicine, Grenada, West Indies; 3 Department of Pediatrics, Texas Tech University Health Sciences Center Amarillo; 4 Neurosurgery, Complex Spine, Swedish Neuroscience Institute; 5 Neurosurgery, Seattle Science Foundation

**Keywords:** dalrymple, ophthalmology, eye anatomy, dalrymple's sign, multiple myeloma, bence jones

## Abstract

Having authored two major ophthalmology textbooks and honored with the eponym, the “Dalrymple sign,” many are familiar with the works of Dr. John Dalrymple when it comes to the eye. However, few are aware of his other, numerous and wide-ranging contributions to the fields of science and medicine. In this article, we discuss the life and work of a man dedicated to the pursuit and advancement of knowledge and education.

## Introduction and background

Many are familiar with the works of Dr. John Dalrymple when it comes to the eye because he was one of the pioneers of medical education within the field of ophthalmology. Having authored two beloved and well-referenced textbooks, "The Anatomy of the Human Eye" and "The Pathology of the Human Eye," Dalrymple is remembered as an expert in his field and is commemorated with the eponym, “Dalrymple’s sign,” used to describe the exophthalmos seen in Grave’s disease [1]. However, few are aware of Dalrymple’s other, numerous contributions to the fields of science and medicine. Though officially given the title of surgeon in the field of ophthalmology, Dalrymple’s works reflect a man who worked not only as a surgeon, but also as a pathologist, biologist, histologist, and artist. His study subjects ranged widely from fish to humans, and he has authored titles such as "Description of an infusory animalcule," "On the structure and function of the human placenta," and "On the nature of ossification in encysted tumor" [2-5]. Within the field of medicine, he was involved in the investigation of a variety of pathologies such as hydroceles, multiple myeloma, dermatology, and more [6-12].

Dalrymple was very active in the scientific and medical communities, frequently lending his expertise to colleagues, participating in several scientific societies, and even, at times, invoking heated debates among his peers. In this article, we discuss Dalrymple’s life and his many contributions.

## Review

### Early life

Dalrymple was born in the city of Norwich in 1803 as the eldest son of Dr. William Dalrymple, a surgeon. John followed in his father’s footsteps, attended Edinburgh University, and became a member of the Royal College of Surgeons in 1827. After settling in London and designating ophthalmology as his field of choice, Dalrymple was elected assistant-surgeon at the Royal London Ophthalmic Hospital in 1832. Two years later, Dalrymple published his first book, *The Anatomy of the Human Eye*—a 300-page account of the history and current knowledge of the eye, with five plates of illustrations and accompanying explanations [1,13-14].

Within the book preface, Dalrymple explains his reason for writing the book [14]:

“During this period, I delivered to the pupils of the London Ophthalmic Infirmary a course of lectures upon the anatomy of the human eye, accompanied by a series of dissections laboriously and minutely followed out. For the purposes of these lectures many authors were of course consulted, and the knowledge contained in their respective works selected and condensed. In my search for authorities, I found no work in the English language, especially dedicated to the anatomy of the organ of vision…”

“…very numerous monographs on separate and particular structures of that organ are to be found, either published in distinct form, or scattered through our scientific journals. But written at very different periods, and almost wholly without system or arrangement, it is difficult, at least for students to avail themselves of the valuable treasures they contain. Under these circumstances, I have been induced to combine in a monographic form the many discoveries and improvements that have of late years enriched the department of anatomical sciences.”

### Medical career and publications

In the following years, Dalrymple published several scientific articles and became a full surgeon. Scientific publications during this time clearly refer to Dalrymple as an active member within the scientific community. As an avid researcher, Dalrymple frequently published original articles and letters to the editor in response to various new developments [15-18]. As evidenced by the preface of his book, Dalrymple believed in the spirit of collaboration and in advancing education. Several papers published by his peers during this time either cite his publications or mention his name and work within the article as a collaborator [10,19-20].

As is typical within any lively community, conflicts often arise. In one instance, Dalrymple incited a heated exchange with Dr. WC Wallace of New York because Dalrymple implied that the idea for Wallace’s paper was taken from his demonstration [21]:

“In a number of the American Journal of Science and Arts, will be found a somewhat similar account of a muscle, discovered in the eye of the streaked bass…by Mr. W. Clay Wallace, surgeon to the New York institution for the blind. This gentleman did me the favor to send me over, about twelve months since, his paper published in that journal. From the circumstance of my not being aware of being personally acquainted with Mr. Wallace, I cannot help suspecting that he is one of the Americans to whom the observations made by me, were imparted at the ophthalmic hospital, some years ago. I beg, however, to return him my thanks for much new and interesting matter, which has been added by him.”

Wallace did not take kindly to this accusation and wrote a rebuttal to Dalrymple’s letter [22]:

“I am not an American, therefore the gentleman thus roundly accused are fully exculpated. As there was nothing on the subject in Mr. Dalrymple’s book, a copy of my paper was forwarded to him soon after his publication appeared. If disposed to deviate so far from candour and courtesy as he has done, I might say, with far more plausibility, I cannot help suspecting that is one of those to whom the observations made by me were imparted through the medium of ‘Silliman’s Journal’ in 1834, and now published as his own in the ‘Magazine of Natural History’ in 1838… I have demonstrated the way in which the eye is adjusted to distances in different animals, to many gentlemen of the highest rank in the profession, and there was no difference of opinion about the structure and uses of the various parts of the organ. An attentive examination is all that is required to make the adjustment of the eye as plain as any other truth in physiology.”

Upon reading Wallace’s rebuttal, Dalrymple responded by insisting on the originality of his work and apologizing for his accusation [23]:

“…Allow me space for a few observations, in order that I may put myself right with regard to the somewhat angry remarks of Mr. Clay Wallace, of New York… That I should, however, have conceived that Mr. Wallace, residing in, and attached to one of the public establishments of, New York, was an American either by birth or adoption, was at least a very pardonable mistake, and one that involved him no disgrace. He has, however, retaliated on me by suspecting that I had read and copied, without acknowledgement, the observations made by him in Silliman’s Journal in 1834. My answer is that I have never seen that Journal, nor did I know anything of Mr. Wallace’s dissections… And further, that my dissections were made two years or more previous to the date of Mr. Wallace’s paper in Prof. Silliman’s Journal.

…as I find Mr. Wallace has distinctly denied, in a recent number of Silliman’s Journal, having either seen or heard of my observations, I am bound in justice to relieve him from any imputation, which I am now sorry I have made. While anxious to clear myself from the suspicion of plagiary, I can do no less than withdraw that portion of my charge which relates to Mr. Wallace…”

Rightly so, Dalrymple was proud of his achievements and did not shy away from claiming credit when it is due. In another letter to the editor, published in the *Lancet* 1841, he discusses the contents of Ferrall’s paper, “On the Anatomy and Physiology of certain Structures in the Orbit not previously described,” and how its claim of a new discovery, “not hitherto been noticed by anatomists,” had already been covered in his book, *The Anatomy of the Human Eye*. Dalrymple went on further to explain [24]:

“The only difference between this description in 1834, and the novelties claimed by M. Bonnet and Mr. Ferrall, is, that I have described the capsule as cellular and they as fibrous; while, upon a careful review of the question, I still maintain that this tunica vaginalis (a term I have no objection to) is composed of condensed cellular membrane, and by no means a true fibrous tissue in the ordinary sense of the word. It is a little too much fashion of the present day to forget the labours of those who have gone before, and that custom lends force to the old adage, ‘Omnis noveltas nil nisi oblivion.’”

As evidence of Dalrymple’s reputation, the editors responded to his letter by praising Dalrymple’s book and being inclined toward Dalrymple’s claims [24].

Dalrymple was undoubtedly even more involved in the fields of medicine than his publications reflect, as papers during this time seem less stringent about correctly acknowledging and citing collaborators. Several papers mention Dalrymple’s name within the text, yet provide little detail on his exact contributions [25-26].

Perhaps one of the most unique contributions that Dalrymple offered was his drawings. There are numerous examples of his works in various books and journal articles. These intricate pieces reflect his skill as a microscopist and his patience and dedication toward the advancement of science.

In 1846, Dalrymple’s collaboration with Dr. Henry Bence Jones elucidated the skeletal defects found in patients with multiple myeloma. In “On the microscopial character of mollities ossium,” Dalrymple conducted and documented the post-mortem examination of Mr. Thomas Alexander McBean, the first well-documented case to be diagnosed with multiple myeloma [7,10]. Examining the patient’s two lumbar vertebrae and a rib, Dalrymple remarked on the demineralized characteristic of the bones and described the histology of his cells [9]: 

“The disease appears to have commenced in the cancellated structure of the bone, for the external osseous laminae are firmer and more healthy than the internal. The smooth surface of the rib, however, is raised by internal growths elevating the outer laminae here and there, into irregularly-sized and rounded dark red projections, visible through the periosteal covering. The outer layers are still hard, requiring the exertion of some force to cut them; they are thin, however, and when sliced, expose large cancellous cavities filled by a red gelatiniform substance, threaded here and there by fine bony fibres.” 

Having accomplished so much in his career, it is hard to believe Dalrymple was chronically plagued by ill health. Dalrymple was sick so often that he frequently could not attend his surgeries, and in 1847, an additional assistant surgeon was hired specifically to assist with his workload [1]. Finally, in 1849, Darymple retired from his position at the Royal London Ophthalmic Hospital. Ironically, he was immediately appointed as Consulting Surgeon of North London Infirmary; however, the hours of this new position were much better-suited for his condition. In 1850, he was elected as a Fellow of the Royal Society Member and the following year, as a member of the Council of the Royal College of Surgeons of England [1,13].

### Later life

Having had poor health his entire life, Dalrymple passed away on May 2, 1852, at the age of 49, due to renal disease [1,13]. His second book, *The Pathology of the Human Eye*, was published just before his death. The book contained 36 watercolor paintings of the eye by various artists [13]. Before its publication, the fasciculus was well-received, with reviews praising its beautiful artwork and the variety of pathologies documented: In the *Edinburgh Medical and Surgical Journal *[27]:

“In these engravings, Mr. Dalrymple undertakes to illustrate by coloured drawings the principal disease of the eye and its appendages. He does not profess, he states, to give a systematic treatise on the subject, but to illustrate in a series of drawings, the various forms of ophthalmic disease as they occur in nature with such explanations as may adequate to identify them with the symptoms, and with the general treatment of the case…

…Upon the whole, these drawings possess qualifications which recommend them strongly to the attention of the surgeon.

In the *Provincial Medical and Surgical Journal* [28]:

“…Mr. Dalrymple has, we think, judged rightly in throwing the results of his experience into the present form. His work thus becomes illustrative of any one or all of these systematic treatises, and indeed makes frequent reference to many of them for details of symptoms and treatment; while, through its admirable couloured drawings, it at once brings the objected symptoms prominently forward, and characterizes and defines them in the accompanying text in such a manner as to render the work most valuable for study and consultation…

…Of the manner in which the drawings are executed and coloured, it is scarecely possible to speak too highly; they are beautiful and characteristic represenations of the diseases which they illustrate, and the entire work, if completed in the same style of excellence, cannot fail to give the highest satisfaction to those who may become possessed of it.”

In the *British and Foreign Medical Chirurgical Journal* [29]:

“The fasciculus of Mr. Dalrymple’s ‘*The* *Pathology of the Human Eye*’ now before us, is perhaps the most interesting that has yet appeared, as regards alike the subjects treated, and the mode in which they are handled, both by the author, and the artists who have executed his illustrations …”

The eponym “Dalrymple’s sign” is likely to have originated after the publication of the book, *The Pathology of the Human Eye*, as an exhaustive search of all of Dalrymple’s published works resulted in no separate accounts or case reports on this topic. In 1849, Cooper published “On protrusion of eyes,” mentioning Dalrymple’s extensive experience and pathophysiologic explanation for this ophthalmologic sign. In this paper, there is no use of the eponym either, lending evidence to the fact that it manifested later, possibly after Dalrymple’s death. Cooper offers Dalrymple’s explanation in the following excerpt:

“The prominence of the eyes is probably due to the operation of two causes. An absence of the proper tonicity of the muscles by which the eyes are retained in their natural positions in the orbit; and some amount of venous congestion of the tissues forming the cushion behind the globes.” [19]

In his final will and testament, Dalrymple, staying true to his role as an educator, bequeathed his drawings of diseases of the eye for the use of the students at the Royal Ophthalmic Hospital. Per James’ biography of Dalrymple, over 70 years after Dalrymple’s death, “Those beautiful paintings of diseases of the eyes are still preserved in the library of the Royal London Ophthalmic Hospital” [1].

## Conclusions

In his relatively short career, John Dalrymple contributed greatly to the fields of science and medicine. Having authored two of the earliest and most comprehensive texts on ophthalmology, as well as numerous journal articles, Dalrymple is well-deserving of an eponym to honor his work. Had he lived a longer, healthier life, Dalrymple would have undoubtedly produced further works of art and scientific advancements.
